# Supplementary material for: Impact of COVID-19 on antenatal care provision at public hospitals in the Sidama region, Ethiopia: A mixed methods study
Source: PLoS One. 2024 Apr 18;19(4):e0301994. doi: 10.1371/journal.pone.0301994 (PMC11025829; doi:10.1371/journal.pone.0301994)
Supplement: S2 Table — (DOCX) [file pone.0301994.s002.docx]

S2 Table: Sociodemographic characteristics of study participants (N=28).

| No | Six | Age in years | Qualification | Work experience in years |
| --- | --- | --- | --- | --- |
| 1 | Female | 26 | BSc midwife | 4 |
| 2 | Female | 30 | BSc midwife | 8 |
| 3 | Female | 26 | BSc midwife | 8 |
| 4 | Female | 26 | BSc midwife | 5 |
| 5 | Female | 28 | BSc midwife | 7 |
| 6 | Female | 28 | BSc midwife | 5.7 |
| 7 | Female | 29 | BSc midwife | 4 |
| 8 | Female | 34 | BSc midwife | 9 |
| 9 | Female | 34 | BSc midwife | 12 |
| 10 | Female | 35 | BSc midwife | 10 |
| 11 | Female | 25 | Diploma midwife | 4 |
| 12 | Female | 27 | Diploma midwife | 5.8 |
| 13 | Female | 45 | BSc midwife | 12 |
| 14 | Male | 25 | BSc midwife | 4 |
| 15 | Female | 28 | BSc midwife | 5 |
| 16 | Male | 34 | IESO | 5 |
| 17 | Male | 33 | IESO | 4 |
| 18 | Female | 28 | Obs/Gyn R3 | 3 |
| 18 | Male | 31 | Obs/Gyn R4 | 2 |
| 20 | Male | 36 | Obs/Gyn R4 | 1 |
| 21 | Male | 33 | Obs/Gyn R4 | 2 |
| 22 | Male | 32 | Obstetrician/gynaecologist | 2.5 |
| 23 | Male | 32 | Obstetrician/gynaecologist | 3 |
| 24 | Male | 32 | Obstetrician/gynaecologist | 3 |
| 25 | Male | 30 | Obstetrician/gynaecologist | 0.6 |
| 26 | Male | 33 | Obstetrician/gynaecologist | 4 |
| 27 | Male | 34 | Obstetrician/gynaecologist | 4 |
| 28 | Male | 45 | Obstetrician/gynaecologist | 20 |

NB: IESO: Integrated emergency surgical officer.
