# Supplementary material for: Impact of COVID-19 on antenatal care provision at public hospitals in the Sidama region, Ethiopia: A mixed methods study
Source: PLoS One. 2024 Apr 18;19(4):e0301994. doi: 10.1371/journal.pone.0301994 (PMC11025829; doi:10.1371/journal.pone.0301994)
Supplement: Attachment — Submitted filename: Comments.docx [file pone.0301994.s003.docx]

**Comments**

Thank you for your invitation to review this paper. Here are comments listed below:

**Title**

The study is already over searched, Even a systematic review of metanalaysis have been done in Ethiopia entitled with **<**Impact of COVID-19 pandemic on utilization of essential maternal healthcare services in Ethiopia: A systematic review and metaanalysis> or link <https://journals.plos.org/plosone/article?id=10.1371/journal.pone.0281260#:~:text=Impact%20of%20COVID%2D19%20pandemic%20on%20ANC%20services,CI%3A%2015.85%2C%2022.76>) . So what you added to your finding?

The title should be rewrite in SMART form meaning that mainly it should clear where (specifically for local reader better to add < Sidam Region, Ethiopia> and when the study done.

**Abstract**

**Methods section**

What is the exact data collection period? What is your sample size? It is not clear to the reader. The author collect data before covid 19 (March 2019 to February 2020), so how to relate the study with covid 19?

**Result**

No information mentioned about the quantitative data in your result. So please include them.

**Conclusion**

Authors should be selective to generalize the whole finding what they have gotten from the finding. It is not clear that why the authors select only three views (ANC access, uptake and provision) for conclusion?

**Keywords**

The author should select MESH terms for key word selection.

**Introduction**

The first sentence needs citation–page 2, line49-50

Please avoid the use of reference repeatedly. E.g. reference 1, 3, etc. repeated more than two times.

Page 3 line 69, insert citation of reference (9) at the end of sentence.

**Methods**

Please Rewrite as <Methods and Materials>

**Setting**

All information presented here are mentioned in the methods section. So please try to merge it or delete one them since in scientific paper writing form no need of redundancy.

What are your study settings/institutions? It is not clearly stated, and try to focus on your study area and selected settings.

**Quantitative methods**

**Setting**

Please rewrite the whole paragraph of this section. No need of writing unnecessary information in study setting like the first sentence you wrote about study design <*We implemented an interrupted time series (ITS) study design to estimate the average 126 changes in ANC provision during the first six months of the COVID-19 pandemic (March to 127 August 2020) at fifteen public hospitals in the Sidama region. ITS is a study design that 128 evaluates the impact of population-level interventions, including policy changes, infection 129 prevention programmes, and any pandemics like COVID-19 that have been implemented at a 130 clearly defined time (33)>.*

Sample size not determined. Why?

**Data processing and analysis**

It lacks focus. It should describe about data entry and analysis, but not data collection methods, data collection procedures etc.

**Qualitative methods**

Please omit redundancy. Your method part is not clear as a whole. You have three section methods in this document (methods, quantitative methods, and Qualitative methods). You should have one comprehensive methods or a max of two sections of methods.

Study participant recruitment and sampling technique

Who is your research assistant? Please clearly mentioned them

What are your study participants? Patient or health care provider

Why the authors used a purposive sampling technique? Is that good to use non probable sampling methods to write scientific *papers important for scientific evidence? In my view it is not acceptable.*

*Please insert this sentence in <Participants provided written informed consent prior to being interviewed*> in Ethical approval and consent section.

What about Study participant recruitment and sampling technique of *quantitative methods??*

**Data collection tools and procedure**

No information mentioned here that describe about the data collection instrument/ tool you used in measurable way. Where the questionnaire you adapted or adopted? Is the too you used is valid or not? If valid what is its validity and reliability test value?

**Data processing and analysis**

It seems only qualitative study design study. What about Data processing and analysis of quantitative section?

**Results**

Trends in antenatal care provision in fifteen hospitals

Where you have got IRR 1.1%? Please revise it (1%). Line 227

**Qualitative results**

Please rewrite the participants’ response in quotation form.

Where is the result of the quantitative part? You have mentioned only the qualitative and joint result. Please add the missed section.

**Discussion**

Try to discuss both the quantitative and qualitative findings.

Please revise and rewrite in two side comparative form.

**Conclusion**

Replaced conclusion with < conclusions and recommendations>

Try to focus on your finding

e.g. You recommended <*Additional studies are needed to understand the long-term impact the ongoing war in the country on these factors*>. It is out of your scope of study/title.

As a whole please try to contact English language expertise and revise the grammar, punctuation, syntax etc. of the entire document to be clear, easily understandable to the readers.
