# Supplementary material for: Impact of COVID-19 on antenatal care provision at public hospitals in the Sidama region, Ethiopia: A mixed methods study
Source: PLoS One. 2024 Apr 18;19(4):e0301994. doi: 10.1371/journal.pone.0301994 (PMC11025829; doi:10.1371/journal.pone.0301994)
Supplement: Attachment — Submitted filename: Authors response to reviewers.docx [file pone.0301994.s004.docx]

Dear reviewers,

Thank you for taking the time to review our manuscript and for your constructive comments and suggestions, which are vital to improving the quality of the manuscript. We addressed your comments point by point, provided clarifications, and incorporated your suggestion into the manuscript as follows:

| **Reviewer 1 comments** | **Author response** |
| --- | --- |
| Thank you for the good work.  1. the first theme is described as "the impact of ANC access"........I hope it is a typing error and better to write it as" access to ANC or the impact of COVID-19 on ANC access...." | Thank you for your comment, the revision has been made in line 52, page 3.  The impact of COVID-19 on ANC access |
| 2. please remove the last row in Table 3 (Joint display of quantitative, qualitative and mixed methods results ...) | We understand your concern; however, the last row in Table 3 shows the argument about whether qualitative findings support the quantitative findings and, if yes, how and if not, why. We modified the arguments and rigorously explained the confirmation and disconfirmation between quantitative and qualitative findings in Table 3. |
| **Reviewer 2 comments** | **Author response** |
| 1. The study is already over-researched, even a systematic review of meta-analysis has been done in Ethiopia entitled with **<**Impact of COVID-19 pandemic on utilisation of essential maternal healthcare services in Ethiopia: A systematic review and meta-analysis> or Link  <https://journals.plos.org/plosone/article?id=10.1371/journal.pone.0281260>  So, what you added to your finding? | Authors response  Dear reviewer, we appreciate your concern regarding the over-researched issue related to our study. To the best of our search and knowledge, no study is exploring the impact of COVID-19 on ANC access, uptake, and provision in a rigorous method. Included studies in this systematic review and meta-analysis (the link you provided) and other studies used the impact of COVID-19 on ANC utilisation; however, they are quite different. The detailed difference between our study and other studies conducted in Ethiopia is as follows:  1. Most of the studies do not show ANC access, uptake, and provision before and during the pandemic. However, they showed ANC uptake during the pandemic without comparing it to before the pandemic, and they did not explore the impact of COVID-19 on ANC access, uptake, and provision.  2. There are also methodological differences; the current study differs from previously published studies on the analysis model employed in the quantitative study and integration of quantitative and qualitative findings. Hence, previously published studies employed a binary logistic regression model, Paired t-test and non-parametric (Wilcoxon signed-rank) tests to estimate the trends of ANC uptake before and during the pandemic. However, the current study used interrupted time series analysis to estimate the trend of ANC uptake before and during the pandemic based on the recommendations from three biostatisticians. As described in the methods section of this paper, the rationale for using the interrupted time series analysis is found in lines 148-151 of page 6. Interrupted time series analysis is a robust statistical analysis method that recommends evaluating the impact of population-level interventions, including policy changes, infection prevention programmes, and pandemics like COVID-19 that have been experienced at a clearly defined time.  3. As described in lines 154-155, page 6, we used the Poisson regression model because the outcome of interest is count data. Furthermore, in the current study, we controlled seasonal and other incidents' effects during the analysis.  4. We used Braun and Clarke’s thematic analysis approach using NVivo 12 plus, which followed six phases to identify themes in our qualitative study. This has not been conducted in previously published articles from Ethiopia.  5. Our qualitative findings explored the impact of COVID-19 on ANC access, uptake, and provision in a rigorous method, while previous studies did not rigorously explore the impact of COVID-19 on ANC access, uptake, and provision independently.  6. We integrated the quantitative and qualitative findings in a joint display analysis based on the recommendations from John W. Creswell’s book (2014) and Fetters MD (2019), which showed three types of integration for concurrent mixed methods- study data transformation, joint display technique and side-by-side (weaving) in the discussion section. In this study, we used the joint display approach, presented in Table 3; however, previous studies do not indicate which integration approach was used in concurrent mixed methods study.  7. In summary, the above justifications clearly show the difference between our current study from previously published works in the same area in Ethiopia. This study contributes to the body of evidence about critical issues and how stakeholders can maintain and enhance ANC access, uptake, and provision for future health systems in crisis related to the epidemic, pandemic, and any natural and man-made disaster. Additionally, this study adds to the body of knowledge and aims to contribute vital evidence to inform the planning and management of future population-level disasters (including epidemic/pandemic situations). |
| 2. The title should be rewrite in SMART form meaning that mainly it should clear where (specifically for local reader better to add < Sidam Region, Ethiopia> and when the study done. | Thank you for your comment, the revision has been made in line 1, page 1.  Impact of COVID-19 on antenatal care provision at public hospitals in Sidama region, Ethiopia: a mixed method study |
| Abstract  3. Methods section  What is the exact data collection period? | Thank you for your comment, the revision has been made in lines 30-31, page 2.  A concurrent mixed methods study was applied between 14 February 2022 and 10 May 2022 at fifteen public hospitals in the Sidama region. |
| 4. What is your sample size? It is not clear to the reader. | Thank you for your comment. Interrupted time series design focuses on any change or difference in a specific time interval due to interventions such as policy change or any disease outbreak. In our case, monthly ANC uptake significantly differs before and during the pandemic (the pandemic is considered an intervention). In our quantitative study, there is a lack of population-level denominators, and we described it as a limitation. The revision has been made in lines 31-35, page 2.  This study included all pregnant women who uptake ANC before COVID-19 (12 months from March 2019 to February 2020) and during COVID-19 (six months from March 2020 to August 2020) at fifteen public hospitals in the Sidama region. The total number for the ANC1 cohort was 15,150, and 5,850 for the ANC4 cohort. The final dataset amounted to 21,000 women, and 28 maternity care providers were interviewed. |
| 5. The author collect data before covid 19 (March 2019 to February 2020), so how to relate the study with covid 19? | We appreciate your concern, and our objective is to estimate the monthly attendance of women for ANC at public hospitals by comparing before and during the pandemic. This allowed us to assess monthly trends of ANC attendance and control for seasonal and other influencing effects. We extracted data from HMIS at 15 public hospitals for the monthly report before COVID-19 (March 2019 to February 2020) and during COVID-19 from March to August 2020 to estimate monthly trends of ANC uptake before and during the pandemic. As described in lines 38-41, page 2.  We used routinely collected data derived from the health management information system (HMIS) in fifteen hospitals in the Sidama region, Ethiopia. Monthly data were collected from March 2019 to February 2020 (12 months) before COVID-19 and from March to August 2020 (6 months) during COVID-19. |
| 6.Result No information mentioned about the quantitative data in your result. So please include them. | Thank you for your comment, the revision has been made in lines 49-51, pages 2 & 3.  The incidence rate of ANC1 uptake decreased by 0.7% (IRR 0.993, 95%CI 0.990 to 0 .997; P<0.0001) (N =15,150), and ANC4 uptake decreased by 1.8% (IRR 0.982, 95%CI 0.976 to 0.987; P<0.001) (N=5850) in the first six months of the pandemic. |
| 7.Conclusion Authors should be selective to generalize the whole finding what they have gotten from the finding. It is not clear that why the authors select only three views (ANC access, uptake and provision) for conclusion? | We acknowledge your concern, and ANC access, uptake, and provision were identified themes in the qualitative findings and pertinent findings in this study that add new insight to the impact of COVID-19 on ANC provision in this study. Our quantitative and qualitative findings indicated that ANC access, uptake and provision were affected during the pandemic. These three issues (ANC access, uptake, and provision) have been identified as a conduit that affects women attending hospitals for ANC and are essential for policymakers to develop strategies for increasing and sustaining ANC access, uptake, and provision during any future pandemic. Therefore, understanding ANC access, uptake, and provision is crucial for increasing the coverage and quality of ANC, especially in low and low-middle-income countries. |
| 8.Keywords The author should select MESH terms for key word selection | Thank you for your comment, the revision has been made in line 65, page 3.  Keywords: ANC access, uptake, provision, COVID-19, Ethiopia |
| 9. The first sentence needs citation–page 2, line49-50 | Thank you for your comment, the revision has been made in line 68, page 3.  1. Rothan HA, Byrareddy SN. The epidemiology and pathogenesis of coronavirus disease (COVID-19) outbreak. J Autoimmun. 2020; 109:102433. |
| 10. Please avoid the use of reference repeatedly. E.g. reference 1, 3, etc. repeated more than two times. | Thank you for your comment, the revision has been made in lines 69-78, page 3. |
| 11. Page 3 line 69, insert citation of reference (9) at the end of sentence. | Thank you for your comment, the revision has been made in line 88, page 4.  In 2016, the WHO recommended a minimum of eight ANC contacts for all pregnant women (11). |
| Methods 12. Please Rewrite as <Methods and Materials> | Thank you for your comment, the revision has been made in line 109, page 4.  **Methods and materials** |
| 13. Setting  All information presented here are mentioned in the methods section. So please try to merge it or delete one them since in scientific paper writing form no need of redundancy. What are your study settings/institutions? It is not clearly stated, and try to focus on your study area and selected settings. | Thank you for your comment, the revision has been made by merging, deleting, and focusing on our study setting. Described in detail in lines 111-122, page 5.  This study is part of a larger mixed-methods study on the impact of COVID-19 on maternal and perinatal care at public hospitals in the Sidama region of southern Ethiopia. This mixed-methods study was carried out between 14 February 2022 and 10 May 2022 at fifteen public hospitals in the Sidama region. The Sidama region is the 10^th^ newly established region in Ethiopia. The region is currently divided into five city administrations and 31 administrative divisions, known as 'Woredas'. In 2019/2020, the region's total population was 3,983,969, with 1,974,455 males and 2,009,514 females. This region has 928,265 women of reproductive age (15-49), 137,845 of whom gave birth in 2019/2020. This region comprises 14 primary hospitals, three general hospitals, one comprehensive specialised teaching hospital, 123 public health centres, 526 health posts and greater than 108 private clinics. Comprehensive emergency obstetric care (CEmOC) was available in 15 public hospitals in the Sidama region in 2020. |
| **14. Setting**  Please rewrite the whole paragraph of this section. No need of writing unnecessary information in study setting like the first sentence you wrote about study design <We implemented an interrupted time series (ITS) study design to estimate the average 126 changes in ANC provision during the first six months of the COVID-19 pandemic (March to 127 August 2020) at fifteen public hospitals in the Sidama region. ITS is a study design that 128 evaluates the impact of population-level interventions, including policy changes, infection 129 prevention programmes, and any pandemics like COVID-19 that have been implemented at a 130 clearly defined time (33)>. | Thank you for your comment, the revision has been made in lines 132-138, pages 5 & 6.  Data was collected from all fifteen public hospitals that provided CEmOC in the Sidama region. In this study, we included all pregnant women who uptake before COVID-19 (12 months from March 2019 to February 2020) and during COVID-19 (six months from March 2020 to August 2020) at public hospitals in the Sidama region. The first author and research assistants, who have MSc in clinical midwifery, extracted data from fifteen public hospitals HMIS in the Sidama region. |
| 15. Sample size not determined. Why? | Thank you for your comment, the revision has been made in lines 132-136, page 5.  This study included all pregnant women who uptake ANC before COVID-19 (12 months from March 2019 to February 2020) and during COVID-19 (six months from March 2020 to August 2020) at fifteen public hospitals in the Sidama region (the total number of women attending 15 hospitals for ANC from March 2019 to August 2020 was (N=21,000)). |
| **16. Data processing and analysis**  It lacks focus. It should describe about data entry and analysis, but not data collection methods, data collection procedures etc. | Thank you for your comment, the revision has been made in lines 144-146, page 6.  After screening the data, any questions related to data clarity were resolved by revisiting the hospitals and regional health HMIS offices. Data were imported from Microsoft Excel into STATA V.17 for analysis. |
| **Qualitative methods**  17. Please omit redundancy. Your method part is not clear as a whole. You have three section methods in this document (methods, quantitative methods, and Qualitative methods). You should have one comprehensive methods or a max of two sections of methods. | Thank you for your comment, the revision has been made in line 109, page 4 (Methods and materials), line 131, page 5 (data collection methods for quantitative strands), line 143, page 6 (data processing and analysis for quantitative strands), in line 172, page 7 (study approach for qualitative strands), in line 183, page 7,(study participant recruitment and sampling technique for qualitative strands), in line 193, page 7,(data collection tools and procedure for qualitative strands) and in line 207 page 8 (data processing and analysis for qualitative strands). |
| 18. Study participant recruitment and sampling technique | Thank you for your comment, the study participant recruitment and sample technique are described in lines 184-192, page 7.  We explained the purpose of the study to the hospital medical director, chief executive director and maternity care head to get permission to conduct the research. Two research assistants, who have MSc in clinical midwifery, explained in detail the purpose of the study for maternity care providers to volunteer to participate in the interview. We used a purposive sampling technique to recruit staff who provided maternity care before and during the pandemic. Participants gave written informed consent prior to being interviewed. We aimed to recruit approximately 20 participants (10 midwives and 10 obstetricians). Data were saturated at 24 interviews. We conducted another four interviews to confirm that data were saturated prior to ceasing qualitative data collection. |
| 19. Who is your research assistant? Please clearly mentioned them | Thank you for your comment, the revision has been made in line 137, page 5.  Research assistants who have MSc in clinical midwifery and were not included as authorship in this manuscript. The responsibility of research assistants was to facilitate the data collection process, for example, in the study participants’ selection process and facilitate the interviews.  “We” is used in this manuscript for those who are included as authors. |
| 20. What are your study participants? Patient or healthcare provider | Thank you for your comment, the study participants are maternity care providers (midwives, obstetrics and gynaecology residents, integrated emergency surgical officers and obstetricians) described in lines 175-177, page 7. |
| 21. Why the authors used a purposive sampling technique? Is that good to use non probable sampling methods to write scientific papers important for scientific evidence? In my view it is not acceptable. | Thank you for your comment; we appreciate your concern about the scientific evidence generated and using non-probability sampling methods (purposive sampling techniques that we used it). Our study extracted quantitative data from 15 public hospitals' HMISs. Whereas for our qualitative study, we prepared open-ended questions that addressed our objectives, and it was piloted outside of the study area and used purposive sampling techniques. It is not unusual in qualitative research to use a purposive sampling technique as it is necessary to be sure the participants have the appropriate experience to contribute to the data. The qualitative study focuses on the depth of individual experiences and feelings related to our study objective and the health service staff needed to have worked before and during the pandemic. |
| 22. Please insert this sentence in <Participants provided written informed consent prior to being interviewed> in Ethical approval and consent section. | Thank you for your comment, the revision has been made in lines 226, page 9.  Participants provided written informed consent prior to being interviewed. |
| 23. What about Study participant recruitment and sampling technique of quantitative methods?? | Thank you for your comment, and we extracted all ANC1 and ANC4 uptake (N=21000) data from fifteen hospitals' HMISs from March 2019 to August 2020; the data is administrative data (district health information software 2). We described it in lines 132-136, page 5.  This study included all pregnant women who uptake ANC before COVID-19 (12 months from March 2019 to February 2020) and during COVID-19 (six months from March 2020 to August 2020) at fifteen public hospitals in the Sidama region (the total number of women attending 15 hospitals for ANC from March 2019 to August 2020 was (N=21,000)). |
| **Data collection tools and procedure**  24. No information mentioned here that describe about the data collection instrument/ tool you used in measurable way. Where the questionnaire you adapted or adopted?  Is the too you used is valid or not? If valid what is its validity and reliability test value? | Thank you for your comment, develop an interview guide consisting of open-ended questions from different literature and pilot it to check whether it addresses our objectives. We described it in lines 194-198, page 8.  We developed a semi-structured interview guide that consisted of open-ended questions related to the following factors: availability and access to maternal and perinatal care, availability of adopted maternal and perinatal care guidelines related to COVID-19, medical supplies, and skilled health care personnel availability, and how challenges were overcome. The interview guide was piloted with midwives who were not included in this study. |
| **Data processing and analysis**  25. It seems only qualitative study design study. What about Data processing and analysis of quantitative section? | Thank you for your comment, data processing and analysis for quantitative strands are addressed in lines 144-171 on pages 6 &7. |
| **Results**  26. Trends in antenatal care provision in fifteen hospitals  Where you have got IRR 1.1%? Please revise it (1%). | Thank you for your comment, the revision has been made in line 235, page 9.  1% |
| 27. Please rewrite the participants' response in quotation form. | Thank you for your comment, checked the participants' responses written in the quotation form. |
| 28. Where is the result of the quantitative part? You have mentioned only the qualitative and joint result. Please add the missed section. | Thank you for your comment, and we extracted data from HMIS, and the HMIS does not contain some variables, for example, the availability of ANC guidelines, long waiting times, discrimination against those attending the hospital, fear of contracting COVID-19, inadequate PPE, and COVID-19 vaccine hesitancy during the pandemic and we considered it as a limitation in lines 505-509, page 20.  ANC access, uptake and provision were impacted by the unavailability of ANC guidelines, long waiting times, discrimination against those attending the hospital, fear of contracting COVID-19, inadequate PPE, and COVID-19 vaccine hesitancy during the pandemic. Nevertheless, because HMIS could not include these variables, the quantitative part of the study did not address these factors. |
| **Discussion**  29. Try to discuss both the quantitative and qualitative findings.  Please revise and rewrite in two side comparative form. | Thank you for your comment, we used the joint display technique to integrate quantitative and qualitative findings. The integration is shown in lines 477-504, pages 19&20, and Table 3.  The revision has been made in lines 543-548, page 29. |
| **Conclusion**  30. Replaced conclusion with < conclusions and recommendations> | Thank you for your comment, and the revision has been made in lines 617, page 31.  Conclusion and recommendations |
| 31. Try to focus on your finding e.g. You recommended <Additional studies are needed to understand the long-term impact the ongoing war in the country on these factors>. It is out of your scope of study/title. | Thank you for your comments; some of the study participants in the qualitative study mentioned a shortage of medical supplies associated with COVID-19, the country’s internal conflict that could lead to the public hospital providing care for soldiers, and the budget shifting to military logistics supply. Therefore, we recommend additional studies are needed to understand the long-term impact of internal conflict in the country on these factors. |
| 32. As a whole please try to contact English language expertise and revise the grammar, punctuation, syntax etc. of the entire document to be clear, easily understandable to the readers. | Thank you for your comment; the manuscript has been reviewed in detail by three senior authors whose native language is English and an English expert person. Grammarly Premium was used to check the manuscript's spelling, grammar, and punctuation. |
