# Supplementary material for: Impact of COVID-19 on antenatal care provision at public hospitals in the Sidama region, Ethiopia: A mixed methods study
Source: PLoS One. 2024 Apr 18;19(4):e0301994. doi: 10.1371/journal.pone.0301994 (PMC11025829; doi:10.1371/journal.pone.0301994)
Supplement: Attachment — Submitted filename: V 5 Authors response to reviewers_V5.docx [file pone.0301994.s005.docx]

Dear Editorial Office,

Thank you for taking the time to review our manuscript and providing the opportunity to rebut reviewers' and editor's comments. We addressed the reviewers' and editor's comments, and point by point rebuttal of the comments as follows:

| **Reviewer 2 comments** | **Authors rebuttal** |
| --- | --- |
| 1. The study is already over-researched, even a systematic review of meta-analysis has been done in Ethiopia entitled with **<**Impact of COVID-19 pandemic on utilisation of essential maternal healthcare services in Ethiopia: A systematic review and meta-analysis> or Link  <https://journals.plos.org/plosone/article?id=10.1371/journal.pone.0281260>  So, what you added to your finding? | We appreciate the reviewer's concern regarding the over-researched issue related to our study. To the best of our search and knowledge, no study is exploring the impact of COVID-19 on ANC access, uptake, and provision in a rigorous method. Included studies in this systematic review and meta-analysis (the link you provided) and other studies used the impact of COVID-19 on ANC utilisation; however, they are quite different. The detailed difference between our study and other studies conducted in Ethiopia is as follows:  1. Most of the studies do not show ANC access, uptake, and provision before and during the pandemic. However, they showed ANC uptake during the pandemic without comparing it to before the pandemic, and they did not explore the impact of COVID-19 on ANC access, uptake, and provision.  2. There are also methodological differences; the current study differs from previously published studies on the analysis model employed in the quantitative study and integration of quantitative and qualitative findings. Hence, previously published studies employed a binary logistic regression model, Paired t-test and non-parametric (Wilcoxon signed-rank) tests to estimate the trends of ANC uptake before and during the pandemic. However, the current study used interrupted time series analysis to estimate the trend of ANC uptake before and during the pandemic based on the recommendations from three biostatisticians. As described in the methods section of this paper, the rationale for using the interrupted time series analysis is found in lines 140-145 of page 5. Interrupted time series analysis is a robust statistical analysis method that recommends evaluating the impact of population-level interventions, including policy changes, infection prevention programmes, and pandemics like COVID-19 that have been experienced at a clearly defined time.  3. As described in lines 152-155, page 5, we used the Poisson regression model because the outcome of interest is count data. Furthermore, in the current study, we controlled seasonal and other incidents' effects during the analysis.  4. We used Braun and Clarke’s thematic analysis approach using NVivo 12 plus, which followed six phases to identify themes in our qualitative study. This has not been conducted in previously published articles from Ethiopia.  5. Our qualitative findings explored the impact of COVID-19 on ANC access, uptake, and provision in a rigorous method, while previous studies did not rigorously explore the impact of COVID-19 on ANC access, uptake, and provision independently.  6. We integrated the quantitative and qualitative findings in a joint display analysis based on the recommendations from John W. Creswell’s book (2014) and Fetters MD (2019), which showed three types of integration for concurrent mixed methods study data transformation, joint display technique and side-by-side (weaving) in the discussion section. In this study, we used the joint display approach, presented in Table 3; however, previous studies do not indicate which integration approach was used in concurrent mixed methods study.  7. In summary, the above justifications clearly show the difference between our current study from previously published works in the same area in Ethiopia. This study contributes to the body of evidence about critical issues and how stakeholders can maintain and enhance ANC access, uptake, and provision for future health systems in crisis related to the epidemic, pandemic, and any natural and man-made disaster. Additionally, this study adds to the body of knowledge and aims to contribute vital evidence to inform the planning and management of future population-level disasters (including epidemic/pandemic situations). |
| 2. The title should be rewrite in SMART form meaning that mainly it should clear where (specifically for local reader better to add < Sidam Region, Ethiopia> and when the study done. | Thank you for your comment; the revision has been made in line 1, page 1.  Impact of COVID-19 on antenatal care provision at public hospitals in Sidama region, Ethiopia: a mixed method study |
| Abstract  3. Methods section  What is the exact data collection period? | The revision has been made in lines 30-31, page 2.  A concurrent mixed methods study was applied between 14 February 2022 and 10 May 2022 at fifteen public hospitals in the Sidama region. |
| 4. What is your sample size? It is not clear to the reader. | The following revision has been made in lines 25-28, pages 1 and 2:  The total numbers in the ANC1 cohort (at least one ANC contact) and ANC4 cohort (at least four ANC contacts) were 15,150 and 5,850, respectively, forming a combined final dataset of 21,000 women.  As we used data from all the women who accessed ANC from March 2019 to August 2020, there was no need for a sample size calculation. |
| 5. The author collect data before covid 19 (March 2019 to February 2020), so how to relate the study with covid 19? | Our objective was to estimate the monthly attendance of women for ANC at public hospitals by comparing data before and data during the pandemic. This allowed us to assess monthly trends of ANC attendance, and to control for seasonal and other influencing effects. We extracted data from HMIS at 15 public hospitals for the monthly report before COVID-19 (March 2019 to February 2020) (twelve months of data) and during COVID-19 from March to August 2020 (six months of data) to estimate monthly trends of ANC uptake before and during the pandemic, as described in lines 38-35, page 2.  We used an interrupted time series model to estimate trends of monthly ANC uptake in the two periods (before and during COVID-19). Routinely collected monthly data were derived from the hospitals’ health management information system (HMIS) and imported into Stata version 17 for analysis. |
| 6.Result No information mentioned about the quantitative data in your result. So please include them. | The revision has been made in lines 43-44, page 2. Our findings indicate that ANC1 uptake and ANC4 uptake, respectively, decreased by 0.7% and 1.8% in the first six months of the pandemic. |
| 7.Conclusion Authors should be selective to generalize the whole finding what they have gotten from the finding. It is not clear that why the authors select only three views (ANC access, uptake and provision) for conclusion? | We acknowledge the reviewer’s concern, and ANC access, uptake, and provision were identified themes in the qualitative findings and pertinent findings in this study that add new insight to the impact of COVID-19 on ANC provision in this study. Our quantitative and qualitative findings indicated that ANC access, uptake and provision were affected during the pandemic. These three issues (ANC access, uptake, and provision) have been identified as a conduit that affects women attending hospitals for ANC and are essential for policymakers to develop strategies for increasing and sustaining ANC access, uptake, and provision during any future pandemic. Therefore, understanding ANC access, uptake, and provision is crucial for increasing the coverage and quality of ANC, especially in low and low-middle-income countries. |
| 8.Keywords The author should select MESH terms for key word selection | The revision has been made in line 58, page 3.  Keywords: ANC access, uptake, provision, COVID-19, Ethiopia |
| 9. The first sentence needs citation–page 2, line49-50 | The revision has been made in line 61, page 3.  1. Rothan HA, Byrareddy SN. The epidemiology and pathogenesis of coronavirus disease (COVID-19) outbreak. J Autoimmun. 2020; 109:102433. |
| 10. Please avoid the use of reference repeatedly. E.g. reference 1, 3, etc. repeated more than two times. | The revision has been made in lines 64-71, page 3. |
| 11. Page 3 line 69, insert citation of reference (9) at the end of sentence. | The revision has been made in line 81, page 3.  In 2016, the WHO recommended a minimum of eight ANC contacts for all pregnant women. |
| Methods 12. Please Rewrite as <Methods and Materials> | We appreciate the reviewer's concerns; we did not use any materials. Therefore, we put it as “methods.” |
| 13. Setting  All information presented here are mentioned in the methods section. So please try to merge it or delete one them since in scientific paper writing form no need of redundancy. What are your study settings/institutions? It is not clearly stated, and try to focus on your study area and selected settings. | The revision has been made by merging, deleting, and focusing on our study setting. Described in detail in lines 103-114, page 4.  The revision has been made as follows:  This study is part of a larger mixed-methods investigation of the impact of COVID-19 on maternal and perinatal care at 15 public hospitals in the Sidama region of southern Ethiopia, carried out between 14 February and 10 May 2022. Sidama is the 10^th^ newly established region in Ethiopia. The region is currently divided into 5 city administrations and 31 administrative divisions, known as ‘Woredas’. In 2019/2020, the region’s total population was 3,983,969, with 1,974,455 males and 2,009,514 females. Sidama has 928,265 women of reproductive age (15–49), 137,845 of whom gave birth in 2019/2020. Regarding healthcare provision, the region has 14 primary hospitals, 3 general hospitals, 1 comprehensive specialised teaching hospital, 123 public health centres, 526 health posts, and over 108 private clinics. Comprehensive emergency obstetric care was available in 15 public hospitals in Sidama during 2020. |
| **14. Setting**  Please rewrite the whole paragraph of this section. No need of writing unnecessary information in study setting like the first sentence you wrote about study design <We implemented an interrupted time series (ITS) study design to estimate the average 126 changes in ANC provision during the first six months of the COVID-19 pandemic (March to 127 August 2020) at fifteen public hospitals in the Sidama region. ITS is a study design that 128 evaluates the impact of population-level interventions, including policy changes, infection 129 prevention programmes, and any pandemics like COVID-19 that have been implemented at a 130 clearly defined time (33)>. | The revision has been made as follows:  Data were collected from all 15 public hospitals providing comprehensive emergency obstetric care in Sidama at the time of the study. The sample included all pregnant women who attended ANC in the 12 months before the COVID-19 pandemic (March 2019 to February 2020) and during the six months of the pandemic (March to August 2020), totalling 21,000. |
| 15. Sample size not determined. Why? | The following revision has been made in lines 125-131, page 5:  Data were collected from all 15 public hospitals providing comprehensive emergency obstetric care in Sidama at the time of the study. The sample included all pregnant women who attended ANC in the 12 months before the COVID-19 pandemic (March 2019 to February 2020) and during the six months of the pandemic (March to August 2020), totalling 21,000 women. As we used data from all the women who attended ANC from March 2019 to August 2020, there was no need for a sample size calculation. |
| **16. Data processing and analysis**  It lacks focus. It should describe about data entry and analysis, but not data collection methods, data collection procedures etc. | The revision has been made in lines 140-144, page 5.  After screening the data, any questions regarding data clarity were resolved by revisiting the hospitals and regional HMIS offices. Data were imported from Microsoft Excel into Stata version 17 for analysis. We performed an interrupted time series analysis (ITSA) to estimate trends in the uptake of ANC across two periods: before COVID-19 (March 2019 to February 2020) and during COVID-19 (March to August 2020). |
| **Qualitative methods**  17. Please omit redundancy. Your method part is not clear as a whole. You have three section methods in this document (methods, quantitative methods, and Qualitative methods). You should have one comprehensive methods or a max of two sections of methods. | The revision has been made, methods in one section in line 101, page 4  Methods |
| 18. Study participant recruitment and sampling technique | The study participant recruitment and sample technique are described in lines 188-198, page 7.  Within each chosen hospital, we explained the study’s purpose to the hospital medical director, chief executive director, and maternity care head, seeking their permission to conduct the research. Subsequently, two research assistants (both with an MSc in clinical midwifery) explained the study’s purpose in detail to maternity care providers who volunteered to be interviewed. We used purposive sampling to recruit staff who provided maternity care both before and during the pandemic. All participants provided written informed consent prior to being interviewed. We aimed to recruit approximately 20 participants (10 midwives and 10 obstetricians). Data reached saturation at 24 interviews. We conducted another four interviews to confirm that data were saturated before ending qualitative data collection. |
| 19. Who is your research assistant? Please clearly mentioned them | Research assistants who have MSc in clinical midwifery were not included as authors in this manuscript. As they were data collectors, they did not meet the criteria for co-authorship of the paper. The responsibility of research assistants was to facilitate the data collection process, for example, in the study participants’ selection process and facilitate the interviews.  “We” is used in this manuscript for those who are included as authors. |
| 20. What are your study participants? Patient or healthcare provider | As stated in lines 176-177 on pages 6 & 7, the study participants are maternity care providers (midwives, obstetrics and gynaecology residents, integrated emergency surgical officers and obstetricians). |
| 21. Why the authors used a purposive sampling technique? Is that good to use non probable sampling methods to write scientific papers important for scientific evidence? In my view it is not acceptable. | Purposive sampling was used for the qualitative component of this mixed methods study, as per usual practice. We prepared open-ended questions that addressed our objectives, and it was piloted outside of the study area. It is common in qualitative research to use a purposive sampling technique as it is necessary to be sure the participants have the appropriate experience to contribute to the data. The qualitative study focuses on the depth of individual views and experiences related to our study objectives; therefore, the participants need to have worked in the relevant hospital settings before and during the pandemic. The last author on this paper is a senior academic with a PhD and more than a decade’s experience in qualitative methodologies and methods. |
| 22. Please insert this sentence in <Participants provided written informed consent prior to being interviewed> in Ethical approval and consent section. | The revision has been made in lines 234, page 8.  Participants provided written informed consent prior to being interviewed. |
| 23. What about Study participant recruitment and sampling technique of quantitative methods?? | The following revision has been made in lines 125-131, page 5.  Data were collected from all 15 public hospitals providing comprehensive emergency obstetric care in the Sidama region at the time of the study. The sample included all pregnant women who attended ANC in the 12 months before the COVID-19 pandemic (March 2019 to February 2020) and during the six months of the pandemic (March to August 2020), totalling 21,000 women.  We collected data from the medical records of all women who attended ANC from March 2019 to August 2020, therefore, there was no need for recruitment of individuals or for a sample size calculation. In addition, interrupted time series design focuses on any change or difference in a specific time interval due to interventions such as policy change or any disease outbreak. In our case, monthly ANC uptake significantly differs before and during the pandemic (the pandemic is considered an intervention). In our quantitative study, there is a lack of population-level denominators, and we described it as a limitation. |
| **Data collection tools and procedure**  24. No information mentioned here that describe about the data collection instrument/ tool you used in measurable way. Where the questionnaire you adapted or adopted?  Is the too you used is valid or not? If valid what is its validity and reliability test value? | We appreciate the reviewer’s concern; for quantitative data, our objective is to determine whether COVID-19 impacts ANC uptake or not. However, for qualitative data, we developed an interview guide consisting of open-ended questions from different literature and piloted it to check whether it addresses our objectives. A qualitative study does not assess internal consistency (to calculate Cronbach alpha) like a quantitative questionnaire.  This is described in lines 200-205, page 7.  We developed a semi-structured interview guide comprising open-ended questions concerning the following factors: availability of and access to maternal and perinatal care; availability of adopted maternal and perinatal care guidelines related to COVID-19; availability of medical supplies and skilled healthcare personnel; and how challenges were overcome. The interview guide was approved by Ethics Committees in Australia and Ethiopia, and the interviews were piloted with midwives who were not included as participants in this study. |
| **Data processing and analysis**  25. It seems only qualitative study design study. What about Data processing and analysis of quantitative section? | Quantitative data processing and analysis are addressed in lines 140-171 on pages 5 &6. |
| **Results**  26. Trends in antenatal care provision in fifteen hospitals  Where you have got IRR 1.1%? Please revise it (1%). | The revision has been made in line 243, page 9.  1% |
| 27. Please rewrite the participants' response in quotation form. | The participants' responses are written in quotation form, indented and italicised, with quotation marks. |
| 28. Where is the result of the quantitative part? You have mentioned only the qualitative and joint result. Please add the missed section. | We appreciate the reviewer’s and editors’ comments, the HMIS from which we extracted the data does not contain all of the variables. For example, there were no available quantitative data of relevance to the absence of ANC guidelines, long waiting times, community discrimination against those attending the hospital, fear of contracting COVID-19, inadequate PPE, or COVID-19 vaccine hesitancy. Therefore, we collected the qualitative findings on these subthemes, but they could not be corroborated or contradicted.  We state it as a limitation in lines 531-535, page 18. |
| **Discussion**  29. Try to discuss both the quantitative and qualitative findings.  Please revise and rewrite in two side comparative form. | We acknowledge the reviewer's suggestions, there are three types of data integrations for concurrent mixed methods study: data transforming, joint display technique and side-by-side comparative form of quantitative and qualitative findings. We used the joint display technique in which there is no need to use a side-by-side comparative form in the discussion section. |
| **Conclusion**  30. Replaced conclusion with < conclusions and recommendations> | The revision has been made in lines 617, page 24.  Conclusion and recommendations |
| 31. Try to focus on your finding e.g. You recommended <Additional studies are needed to understand the long-term impact the ongoing war in the country on these factors>. It is out of your scope of study/title. | We acknowledge the reviewer’s concern, some of the study participants in the qualitative study mentioned a shortage of medical supplies associated with COVID-19, the country’s internal conflict that could lead to the public hospital providing care for soldiers, and the budget shifting to military logistics supply. Therefore, we recommend additional studies are needed to understand the long-term impact of internal conflict in the country on these health system factors. |
| 32. As a whole please try to contact English language expertise and revise the grammar, punctuation, syntax etc. of the entire document to be clear, easily understandable to the readers. | Thank you for your comment; the manuscript has been reviewed in detail by two senior authors whose native language is English and an English expert person. Grammarly Premium was used to check the manuscript's spelling, grammar, and punctuation. Additionally, the manuscript was edited and proofread by Elsevier language editing services. |
