# Supplementary material for: Impact of COVID-19 on antenatal care provision at public hospitals in the Sidama region, Ethiopia: A mixed methods study
Source: PLoS One. 2024 Apr 18;19(4):e0301994. doi: 10.1371/journal.pone.0301994 (PMC11025829; doi:10.1371/journal.pone.0301994)
Supplement: Attachment — Submitted filename: PONE-D-23-07658_R2_feedback.docx [file pone.0301994.s006.docx]

Dear Editors and Authors,

Thank you very much for the opportunity, and I appreciate the editors considering me to review this manuscript.

And congratulations to the Authors for this great work. I believe the manuscript is an important study to support existing available evidence on the impact of COVID-19 on maternal health. However, some points need to be elaborated further by the authors to ensure the paper's clarity.

**Feedback:**

1. Introduction: It would be good to explain how COVID-19 in Ethiopia in the introduction
2. Study design and data source: What is HMIS? Please provide its full version
3. Participant recruitment and sampling technique for qualitative data: How many people were approached? And were there any decliners? Please report it.
4. Collection tools and procedure for qualitative data:
   a. How many midwives were involved in the pilot study? And which version of the interview guide was piloted (English/Amharic)?
   b. Were there other people present during the interview besides the interviewer and participant? If yes, please report it.
5. Qualitative data processing and analysis:
   a. How many researchers were involved in the coding and analysis process?
   b. Were participants given a chance to review their transcripts/ summary of findings?
6. The discussion is very long (almost 1500 words), and readers are likely to get lost reading it. I'd highly suggest making it more concise.
7. What about recommendations related to the issue of discrimination and vaccine hesitancy?
