# Supplementary material for: Impact of COVID-19 on antenatal care provision at public hospitals in the Sidama region, Ethiopia: A mixed methods study
Source: PLoS One. 2024 Apr 18;19(4):e0301994. doi: 10.1371/journal.pone.0301994 (PMC11025829; doi:10.1371/journal.pone.0301994)
Supplement: Attachment — Submitted filename: Zemenu Kassa V 1 authors response to reviewers PLOS ONE.docx [file pone.0301994.s007.docx]

Dear Editor and reviewers,

Thank you for taking the time to review our manuscript and for your constructive comments and suggestions, which are vital to improving the quality of the manuscript. We addressed your comments point by point, provided clarifications, and incorporated your suggestion into the manuscript as follows:

| **Editor comments** | **Author response** |
| --- | --- |
| **1.** In the findings section, similar ideas and quotes appear in multiple places. For instance, the community's perception of health facilities as the epicenter of COVID-19 infection is displayed at various points under different themes or sub-themes. | Thank you for your comment; the following revision has been made in lines 335 and 337, page 15. The term ‘Epicentre of COVID-19 infection’ only appears in the ‘Fear of contracting COVID-19’ subtheme. |
| **2.** The same issue arises with transportation-related issues. | Thank you for your comment; the following revision has been made in lines 271 and 275, page 13. The term ‘transport’ only appears in the ‘Shortage of resources’ subtheme. |
| **3.** There is a misalignment between some themes/subthemes and the provided descriptions. For example, in the sub-theme 'Community discrimination against women attending hospital,' the description addresses both community discrimination against women and care providers while the subtheme is about women. | The following revision has been made in line 288, page 13.  Community discrimination against those attending the hospital |
| **4.** The finding section benefit from proper resynthesis. Additionally, the manuscript focuses on the impact of COVID-19 on antenatal care provision. However, unrelated issues regarding vaccine hesitancy are introduced in the findings section without proper context. | Dear editor, we appreciate your concern regarding the relation between ANC and vaccine hesitancy.  Vaccine hesitancy could affect ANC provision, as stated in Table 3.  The qualitative findings demonstrate that COVID-19 vaccine hesitancy impacted ANC provision. The vaccine hesitancy amongst maternity care providers could have increased the reluctance of pregnant women to be vaccinated, since the providers may not have persuaded them of the vaccine’s benefits during pregnancy. Furthermore, vaccine-hesitant maternity care providers’ fear of contracting the virus when providing ANC could have further reduced ANC provision. |
| **5.** The finding section benefit from proper resynthesis. | Thank you for your comment; the qualitative finding synthesis was reviewed iteratively as per the approach of Clarke and Braun in thematic analysis, and modifications have been made across the synthesis of the findings. |
| **Reviewer 3 comments** | **Author response** |
| **1.** Generally, the manuscript need thorough English language edition. The punctuation and grammar need correction to facilitate easy understanding for readers. | Thank you for your comment; the manuscript has been reviewed in detail by two senior authors, both native English speakers and an English expert person. Grammarly Premium was used to check the manuscript's spelling, grammar, and punctuation. Additionally, the manuscript was edited and proofread by Elsevier language editing services. |
| **2.** Is effect or impact appropriate term to indicate results of covid-19 during mentioned times (six months, March to August 2020). | We appreciate your concern; we believe ‘impact’ is an appropriate term for this study. Impact is often used to describe the immediate influence of COVID-19 on ANC. |
| **3.** what was study gaps that authors want to uncover? | The following revision has been made in lines 18 and 19, page 1:  *There is a paucity of studies on the impact of COVID-19 on antenatal care access, uptake, and provision in Ethiopia.* |
| **4.** Did authors calculate minimum sample size? | As stated in lines 131-135, page 5, data were collected from all 15 public hospitals providing comprehensive emergency obstetric care in the Sidama region at the time of the study. The sample included all pregnant women who attended ANC in the 18 months before the COVID-19 pandemic (March 2019 to February 2020) and during the six months of the pandemic (March to August 2020), totalling 21,000 women. As we used data from all the women who attended ANC from March 2019 to August 2020, there was no need for a sample size calculation.  Furthermore, this study is part of a broader mixed methods study, and a minimum sample size was calculated. In fact, it is mandatory to calculate the sample size, so the single population proportion formula was used. The health coverage in the Sidama region 2019/2020 was 77.2%. The level of significance was 5 %(a=0.05), the margin of error was 3 % (d=0.03), and the non-responsive rate was 10%; the final sample was 826. |
| **5.** How authors selected these facilities? | All 15 public hospitals that provided comprehensive emergency obstetrics in the Sidama region were included in the quantitative study. However, as stated in lines 172 -180, pages 6 and 7, four hospitals were selected for the qualitative study. These four public hospitals (including two primary hospitals, one general hospital and one specialised hospital) were chosen for the qualitative study based on the caseload maternity care services provided and the order in which COVID-19 cases were initially reported in the Sidama region. Three different types of hospitals were selected: primary, general, and one specialised hospital that served as a referral centre for the Sidama region and the surrounding population in the Oromia region. This selection allowed for a nuanced understanding of the impact of the pandemic on various tiers of hospitals and their preparedness, response efficiency and the challenges they faced. |
| **6**. How authors selected these participants? | The quantitative data extraction was stated in lines 132-135, page 5:  Data were collected from all 15 public hospitals providing comprehensive emergency obstetric care in the Sidama region at the time of the study. The sample included all pregnant women who attended ANC in the 12 months before the COVID-19 pandemic (March 2019 to February 2020) and during the six months of the pandemic (March to August 2020), totalling 21,000.  For qualitative study, the study participants' selection was described in lines 182 -190, page 7.  Within each chosen hospital, we explained the study’s purpose to the hospital medical director, chief executive director, and maternity care head, seeking their permission to conduct the research. Subsequently, two research assistants (both with an MSc in clinical midwifery) explained the study’s purpose in detail to maternity care providers who volunteered to be interviewed. We used purposive sampling to recruit staff who provided maternity care both before and during the pandemic. All participants provided written informed consent prior to being interviewed. We aimed to recruit approximately 20 participants (10 midwives and 10 obstetricians). Data reached saturation at 24 interviews. We conducted another four interviews to confirm that data were saturated before ending qualitative data collection. |
| **7.** How authors maintained data quality? | Thank you for your comment; the quantitative data quality was maintained by revisiting the hospitals and HMIS offices to address any missing data in the provided Excel spreadsheet, as stated in lines 142 and 143, page 5. Data cleaning and preprocessing were done to check missing data. In addition, based on the study objective, the authors selected robust methods of analysis, which is an interrupted time series analysis through a poison regression model.  The interview guide was approved by the Ethics Committees at UTS HREC and IRB Hawassa University.  For the qualitative study, the data quality was maintained by preparing a study guide and conducting a pilot study, as stated in line 197, page 7:  *The audio recordings were transcribed immediately and listened to iteratively. Simultaneously, bilingual researchers transcribed and translated transcripts into English to check consistency. The transcriptions were imported into NVivo software (QSR International, version 12 Plus) to manage the overall data analysis, as stated in lines 206-209, page 8. All authors reviewed the themes and subthemes in the thematic analysis phases (from coding to writing a report) and approved the final themes, as stated in lines 213 -215, page 8.* |
| **8.** How quantitative data were reported? | The quantitative data were reported based on assumptions of the model (Poisson regression) using a P value less than 0.05 (p<0.05), which is considered statistically significant, and using a 95% confidence interval. The incidence rate ratio (IRR) of ANC1 and ANC4 is reported in the figure, table, and texts, which are stated in lines 150-157, page 6. In addition, we based our reporting on journal submission guidelines.  The mean monthly incidence rate ratio (IRR) of ANC uptake was calculated with a 95% confidence interval (CI) using a Poisson regression model (34, 35) with pre-COVID-19 data as the reference. A Poisson regression model was suitable because the monthly reports of ANC provision comprised count data (non-negative integer values). In ITSA, a Poisson regression model (36) performs better than an autoregressive integrated moving average (ARIMA) model, which is more conventionally used for real-valued time series data. Differences are considered statistically significant at a p-value of less than 0.05 (*p* < 0.05). |
| **9.** Result: line 37 and 38, the sentence is not clear. Was the effect of covid-19 significant? | Thank you for your comment: the revision has been made in lines 40 and 41, page 2:  *Our findings indicate a significant monthly decrease of 0.7% in ANC1 and 1.8% in ANC4 during the first six months of the pandemic.* |
| **10.** Line 41 subtheme ‘COVID-19 vaccine hesitancy’ was identified by authors. Was there a vaccine during specified time? I recommend these subthemes categorized under their themes, so that effect of covid-19 seen boldly. | We understand your concern: the data were collected between 14 February and 10 May 2022. During the data collection, COVID-19 vaccinations were available. Some study participants reported that they did not get vaccinations, for a variety of reasons. These factors could potentially have disrupted the provision of ANC. Further details are provided in Table 3.  The qualitative findings demonstrate that COVID-19 vaccine hesitancy impacted ANC provision. Such hesitancy in maternity care providers could have increased the reluctance of pregnant women to be vaccinated, since the providers would not attempt to persuade them of the vaccine’s benefits during pregnancy. Furthermore, vaccine-hesitant maternity care providers’ fear of contracting the virus when providing ANC could have further reduced ANC provision. |
| **11.** Conclusion and recommendation: is qualitative or quantitative result support this conclusion? Or both? Authors did not indicate adequate finding that support their conclusion in result part of the abstract. Are your recommendation supported by your evidence and updated? What is the study implication? | Thank you for your comment; the following revision has been made in lines 51-57, pages 2 and 3, and the conclusion and recommendation are derived from our concurrent mixed methods findings.  *A lack of medical supplies, discrimination against those attending the hospital, and the absence of ANC guidelines for care provision led to disrupted access, uptake, and provision of ANC during COVID-19. To mitigate disrupted ANC access, uptake and provision, ANC clinics should be equipped with medical supplies. It is crucial to maintain rapport between the community and maternity care providers and provide training for maternity care providers regarding the adapted/adopted guidelines during COVID-19 at the hospital grassroots level for use in the current and future pandemics.* |
| **12.** Authors did not indicate study gaps, why this study is needed. What is the significance of this study? | *Thank you for your comment; the revision has been made in lines 97-109, page 4.*  *In the early stage of the COVID-19 pandemic, Ethiopian government and NGOs shifted their focus towards containing the spread of the virus by implementing a range of measures. These measures included declaring a state of emergency, reducing the passenger capacity in public transport by half, imposing a lockdown and encouraging people to stay at home (27). Consequently, the lockdown measures resulted in job losses for many women (28). These factors posed significant challenges to women’s ability to meet their basic needs (28). As a result, the provision of ANC has been and continues to be, impacted by the direct and indirect consequences of COVID-19 (29). However, the existing studies in Ethiopia on the impact of the COVID-19 pandemic have not rigorously explored its impact on maternity care services, specifically ANC access, uptake, and provision. The paucity of studies on the impact of COVID-19 on ANC access, uptake and provision in Ethiopia made it essential to conduct this study to estimate and explore the impact of COVID-19 on the country’s ANC access, uptake, and provision.*  *In addition, most of the studies show in their titles that they have conducted a mixed methods study, but in many cases, it was simply qualitative and quantitative studies. When studies lack integration and do not tell us which techniques of integration were used and applied in the method, result, or discussion section, this is not truly mixed methods. However, in this study, we integrated the quantitative and qualitative findings in a joint display technique based on the recommendations from John W. Creswell’s book (2014) and Fetters MD (2019), which showed three types of integration for concurrent mixed methods- study data transformation, joint display technique and side-by-side (weaving) in the discussion section. In this study, we used the joint display approach, presented in Table 3; however, previous studies do not indicate which integration approach was used.* |
| **13.** Why qualitative study needed? What is the objective of qualitative study? | Dear reviewer, we appreciate your concern regarding the study methods. We applied a concurrent mixed methods study to estimate and explore the impact of COVID-19 on ANC at public hospitals. The quantitative findings estimate the trends of ANC in the first 12 months before COVID-19 and in the first six months of the pandemic. Simultaneously, the qualitative findings explore maternity care providers' experiences and perceptions of ANC provision before and during the pandemic. The objective of this mixed methods study was to explore whether the qualitative findings confirmed or disconfirmed the quantitative findings. |
| **14.** Line 100 what specific design was used for quantitative study? | Thank you for your comment; the revision has been made in lines 124, page 5  *An interrupted time series design was applied for the quantitative component of the study.* |
| **15**. How about a qualitative study? | As stated in line 169, page 6, we adopted an exploratory design to investigate maternity care providers’ views and experiences of the impact of COVID-19 on ANC provision in the Sidama region. |
| **16**. These two terms are broad terms to describe design, authors need to explain specific design they employed for both type of study. | As stated in line 122, page 5, in this study we used a concurrent mixed-methods design. |
| **17**. Line 107, rewrite the subtitle. ‘Data collection method for quantitative data’ The content should also clearly indicate the tool used, who collected and how you collected the data. | *Thank you for your comment; the revision has been made in line 131, page 5:*  *Data collection methods for quantitative data*  *This study is part of a broader mixed methods study, focusing on the outcome variables ANC1, ANC4, and essential medicines. These variables were extracted from the HMIS office in each hospital. The data was obtained from the HMIS office at each hospital in spreadsheet Excel form. Data on maternal and perinatal care spanning 18 months (March 2019 to August 2020) were collected from the HMIS of all 15 hospitals and were merged into an Excel spreadsheet.*  *As stated in lines 135-136, page 5, the first author and research assistants (all with an MSc in clinical midwifery) extracted data from the hospitals’ HMIS.* |
| **18.**How authors interviewed those who have difficulties in speaking official language? | We appreciate your concern; the research assistants explained the study’s objectives for maternity care providers and provided a personal information sheet for those who volunteered to participate in the study. Subsequently, the research assistant arranged the interview schedule, and the first author conducted the interviews. It is noteworthy that during these interviews, the first author did not encounter any participants facing difficulties with the official language. |
| **19.** Authors failed to explain how they assured quality of data for both quantitative and qualitative methods. | Thank you for your comment; the quantitative data quality was maintained by revisiting the hospitals and HMIS offices to address any missing data in the provided Excel spreadsheet, as stated in lines 142 and 143, page 5. Data cleaning and preprocessing were done to check missing data. In addition, based on the study objective, the authors selected robust methods of analysis, which is an interrupted time series analysis through a poison regression model.  The interview guide was approved by the Ethics Committees at UTS HREC and IRB Hawassa University.  For the qualitative study, the data quality was maintained by preparing a study guide and conducting a pilot study, as stated in line 197, page 7.  The audio recording was iteratively listened to and transcribed. Simultaneously, bilingual researchers also transcribed and translated to English to check consistency. The transcriptions were imported into NVivo software (QSR International, version 12 Plus) to manage the overall data analysis, as stated in lines 206-209, page 8. All authors reviewed the themes and subthemes in the thematic analysis phases (from coding to writing a report) and approved the final themes, as stated in lines 213 - 215, page 8. |
| **20.** How many was the response rate? Was there any missing data? How it was managed? | The quantitative data which used in this study is a routine monthly report on administrative data, and it did not experience any non-response rate. Revisiting the hospitals and HMIS offices to address any missing data in the provided Excel spreadsheet. |
| **21.** Change the following subtitle ‘Qualitative results’ to appropriate subtopic. Check its content and choose. | Thank you for your comments, the revision has been made in line 244 and 245, page 11.  *Qualitative results: Clinicians views and experiences of the impact of COVID-19 on antenatal care provision* |
| **22.** The authors are not expected to write ‘quote’ for each paragraphs. Too many quote indicate poor analysis of the data. | We appreciate your concern; In our synthesis of findings, we limit each paragraph to a maximum of two block quotes. It is worth noting that scholars recommend including at least 40 words in block quotes. The third and last author are experienced and widely published qualitative researchers. |
| **23.** Authors should focus on the major findings. This discussion is not easy to understand and focused. | Thank you for your comment; the revision has been made in the discussion section in lines 513-655, pages 26-30. We focused on the pertinent findings. |
| **24.** Is the disruption is still there? Authors should cautiously conclude and recommend based on their findings. It may contribute to knowledge of what happened during covid-19. Otherwise, I don’t think this conditions are still there in Sidama. | We appreciate your concern; the conclusion and recommendation derived from the impact of the pandemic on ANC access, uptake, and provision and as a lesson learned for preparing any future health system in crisis. |
| **Reviewer 4 comments** | **Authors response** |
| **1.** Introduction: It would be good to explain how COVID-19 in Ethiopia in the introduction | Thank you for your comment; the revision has been made in lines 97-109 page 4. |
| **2.** Study design and data source: What is HMIS? Please provide its full version | Thank you for your comment; the revision has been made in line 123, page 5:  *health management information system (HMIS)* |
| **3.** Participant recruitment and sampling technique for qualitative data: How many people were approached? And were there any decliners? Please report it. | As stated in line 189 and 191, page 7, we aimed to recruit approximately 20 participants (10 midwives and 10 obstetricians). Data reach saturation at 24 interviews. We conducted another four interviews to confirm that data were saturated before ending qualitative data collection. Fortunately, there were no study participants who withdraw from the interviews. |
| **4.** Collection tools and procedure for qualitative data:  **a.** How many midwives were involved in the pilot study? And which version of the interview guide was piloted (English/Amharic)? | Thank you for your comments, 4 midwives were involved in the pilot study in line 197, page 7. The Amharic version of interview guide was piloted.  The interview guide was approved by Ethics Committees in UTS HREC and IRB Hawassa University. |
| **4b.** Were there other people present during the interview besides the interviewer and participant? If yes, please report it. | Thank you for your comments, there were research assistants who facilitated the interviews, already mentioned in the participant recruitment and sampling technique for qualitative data section line 185 page 7. |
| **5.**Qualitative data processing and analysis:  **a**. How many researchers were involved in the coding and analysis process? | Thank you for your comments, the coding and analysis were conducted by the first author under the supervision of three senior authors, who approved the coding, subthemes, and themes in each step of the thematic analysis using NVivo software (QSR International, version 12 Plus). |
| **5b.** Were participants given a chance to review their transcripts/ summary of findings? | Thank you for your comment, the first author collected data in Ethiopia, and upon completing the data collection, relocated to Australia to analyse the data. As the data were collected anonymously, there was no opportunity to provide a summary of findings for study participants. |
| **6.** The discussion is very long (almost 1500 words), and readers are likely to get lost reading it. I'd highly suggest making it more concise. | Thank you for your comment; the revision has been made in the discussion section in lines 513-655, pages 26-30. |
| **7.** What about recommendations related to the issue of discrimination and vaccine hesitancy? | Thank you for your comment; the following revision has been made in lines 51-57, pages 2 and 3, and the conclusion and recommendation are forwarded based on both quantitative and qualitative findings.  *A lack of medical supplies, discrimination against those attending the hospital, and the absence of ANC guidelines for care provision led to disrupted access, uptake, and provision of ANC during COVID-19. To mitigate disrupted ANC access, uptake and provision, ANC clinics should be equipped with medical supplies. It is crucial to maintain rapport between the community and maternity care providers and provide training for maternity care providers regarding the adapted/adopted guidelines during COVID-19 at the hospital grassroots level for use in the current and future pandemics.* |
