# Supplementary material for: Impact of COVID-19 on antenatal care provision at public hospitals in the Sidama region, Ethiopia: A mixed methods study
Source: PLoS One. 2024 Apr 18;19(4):e0301994. doi: 10.1371/journal.pone.0301994 (PMC11025829; doi:10.1371/journal.pone.0301994)
Supplement: Attachment — Submitted filename: Zemenu Kassa authors response.docx [file pone.0301994.s008.docx]

Dear Editor,

Thank you for taking the time to review our manuscript and for your constructive comments and suggestions, which are vital to improving the quality of the manuscript. We addressed your comments point by point, provided clarifications, and incorporated your suggestion into the manuscript as follows:

| **Editor comments** | **Author response** |
| --- | --- |
| 1. The second sentence of the conclusion and recommendation section seems better fit into the result section and please move it up. | Thank you for your comment; the following revision has been made in lines 40-44, page 2, as follows:  A lack of medical supplies, fear of contracting COVID-19, inadequate personal protective equipment, discrimination against those attending the hospital, and the absence of antenatal care guidelines for care provision, COVID-19 vaccine hesitancy and long waiting times for ANC led to disrupted access, uptake, and provision of antenatal care during COVID-19. |
| 2. Please don't introduce an abbreviation in the abstract if you are not gong to use it in this section. e.g., HMIS. Your abbreviation use is actually inconsistent throughout the document. For some, you provided multiple full text description (e.g., WHO), while for some others, you started using it without providing a full text discerption at the first use (e.g., ANC). Please make sure your abbreviations use is consistent and follows the standard. | Thank you for your comment; the revision has been made. The abstract has been written without any abbreviation, adhering to the prescribed wordage.  The revision has been made in the introduction section, line 77, page 3 as follows:  antenatal care (ANC) |
| 3. In the abstract section, you stated that you collected 12 months' data before the occurrence of COVID-19, while in the method section it says 18 months data were collected. Please make sure you are consistent. In addition, please also discuss the appropriateness of comparing 12 or 18 months data (before COVID-19) with 6 months data (during COVID-19). | Thank you for your comment; the revision has been made in line 135, page 5.  Twelve months before the COVID-19 pandemic (March 2019 to February 2020).  In addition, we aimed to assess the ANC uptake during the same period initially (March to August 2019 before COVID-19) and March to August 2020 during COVID-19 to control seasonal effects. After consulting a biostatistician, we decided to include the period from September 2019 to February 2020, whether ANC has been affected by other incidences or not, to control other incidents effects as stated in lines 140-142, page 5. This study aims to assess any monthly changes in ANC1 and ANC4 attendance before COVID-19 (March 2019 to February 2020) and during COVID-19 (March to August 2020). The study was conducted while controlling seasonal effects and other incidents impacting ANC1 and ANC4 attendance. Therefore, this study compared monthly changes in ANC1 and ANC4 attendance before COVID-19 (March 2019 to February 2020) and during COVID-19 (March to August 2020). |
| 4. Please move table 2 to supplementary file. | The revision has been made by moving Table 2 to Supplementary Table 2. |
| 5. Under the community discrimination against those attending the hospital sub-theme, you clearly discussed about the community discrimination against those attending the hospital. However, you gave little emphases on its link to the access to ANC. You have tried to say a few things in a second sentence of this section, but didn't substantiate it with the data from the participants. Please clearly show the link of this sub-theme to the access to ANC by substantiating it with the data. | Thank you for your comment; the following revision has been made in lines 306 and 317, page 14, as follows:  resulting in limited access to ANC.  Consequently, this discrimination resulted in limited access to ANC. |
| 6. Neither your discerption nor the provided quate still doesn't show the influence of COVID-19 vaccine hesitancy on ANC service utilization. You have tried to respond on the response letter, but the description should appear on the manuscript too. Please make sure that this claim is substantiated with evidence came from the participants. It shouldn't be the authors speculation. | Thank you for your comment; the following revision has been made in lines 432-434, page 18, as follows:  If maternity care providers are hesitant about the COVID-19 vaccine, it may impact pregnant women's confidence in the vaccine's safety and efficacy. This could lead to lower vaccine rates and hinder ANC provision. In addition, at the beginning of the COVID-19 vaccine rollout, priority was given to frontline healthcare workers to enhance medical care, including ANC provision. |
